# *Edwardsiella tarda* Tunes Tricarboxylic Acid Cycle to Evade Complement-Mediated Killing

**DOI:** 10.3389/fimmu.2017.01706

**Published:** 2017-12-07

**Authors:** Zhi-xue Cheng, Qi-yang Gong, Zhe Wang, Zhuang-gui Chen, Jin-zhou Ye, Jun Li, Jie Wang, Man-jun Yang, Xiao-peng Ling, Bo Peng

**Affiliations:** ^1^Center for Proteomics and Metabolomics, State Key Laboratory of Bio-Control, School of Life Sciences, Sun Yat-sen University, Guangzhou, China; ^2^Pediatric Intensive Care Unit, Department of Pediatrics, The Third Affiliated Hospital of Sun Yat-sen University, Guangzhou, China; ^3^Laboratory for Marine Biology and Biotechnology, Qingdao National Laboratory for Marine Science and Technology, Qingdao, China

**Keywords:** *Edwardsiella tarda*, serum resistance, reprogramming metabolomics, the tricarboxylic acid cycle, membrane potential

## Abstract

Evasion of complement-mediated killing is a common phenotype for many different types of pathogens, but the mechanism is still poorly understood. Most of the clinic isolates of *Edwardsiella tarda*, an important pathogen infecting both of human and fish, are commonly found serum-resistant. To explore the potential mechanisms, we applied gas chromatography-mass spectrometry (GC-MS)-based metabolomics approaches to profile the metabolomes of *E. tarda* EIB202 in the presence or absence of serum stress. We found that tricarboxylic acid (TCA) cycle was greatly enhanced in the presence of serum. The quantitative real-time PCR (qRT-PCR) and enzyme activity assays validated this result. Furthermore, exogenous succinate that promotes the TCA cycle increased serum resistance, while TCA cycle inhibitors (bromopyruvate and propanedioic acid) that inhibit TCA cycle, attenuated serum resistance. Moreover, the enhanced TCA cycle increased membrane potential, thus decreased the formation of membrane attack complex at cell surface, resulting serum resistance. These evidences suggested a previously unknown membrane potential-dependent mechanism of serum resistance. Therefore, our findings reveal that pathogen mounts a metabolic trick to cope with the serum complement-mediated killing.

## Introduction

*Edwardsiella tarda* is a Gram-negative intracellular pathogen that belongs to the Enterobacteriaceae family with a broad host range that includes mammals, reptiles, and fish (1–3). *E. tarda* causes acute gastroenteritis, meningitis, septicemia, and wound infections in infected humans (4, 5). The outbreak of edwardsiellosis caused by *E. tarda* in fish represents one of the most severe diseases in farmed fish like in barramundi (*Lates calcarifer*) in different countries, which led to a great economic loss in aquaculture and the fishing industry worldwide in recent years (6–9). Therefore, it is especially emergent to understand bacterial pathogenesis for controlling the infectious disease caused by *E. tarda*.

Although the pathogenic factors in *E. tarda* were not fully elucidated, a huge effect has been made to understand the pathogenesis of *Edwardsiella* species. The infection by this species heavily relies on the pathogenic factors like type III and type IV secretion systems (10–12). As a representative intracellular pathogen, *E. ictaluri* senses the intracellular environment like pH and phosphate concentration, which drive the type III secretion system expression through regulators, EsrA, EsrB, and EsrC (13). In addition, *E. ictaluri* actively modulates the vacuolar pH and secretes urease for intracellular replication in macrophages (14, 15). The type VI secretion system of *E. tarda* injects effectors like EvpP into host cell, which inhibits inflammasome and prevents the downstream pathways, leading to pyrotosis (16).

Besides the virulent factors, serum resistance is another determinant for bacterial persistence inside the host in many Gram-negative and -positive pathogens, including *E. tarda* (17, 18). Complement system is the frontline of innate immune defense system. The activation of complement system leads to the formation of membrane attack complex (MAC) that forms channel on the bacterial membrane, causing bacterial lysis. One of the mechanism for serum resistance is thus to reduce the deposition of MAC on the bacterial membrane (19). In addition, complement system also binds bacteria and opsonizes them for the subsequent phagocytosis and antibody production. Complement system plays critical roles in clearing pathogens through classical pathway, alternative pathway, or lectin pathway (20, 21). Thus, delineating the mechanisms for serum resistance is of great importance for combating serum-resistant pathogens.

Accumulating evidences have indicated that *E. tarda* is an intracellular pathogen with the capacity to evade host immune defense, which is reflected in one aspect that *E. tarda* can survive in host serum (1, 22, 23). Further study indicates that *E. tarda* evades the serum complement-mediated killing by preventing complement activation *via* the alternative pathway, and that heat-labile surface structures likely play an essential role in the complement evasion of *E. tarda* (23). These data provide the basis for further revealing of the detailed mechanisms of complement evasion in *E. tarda*.

Despite the recent progress, the mechanism of serum resistance of *E. tarda* is still unknown. Previous reports on bacterial serum resistance focused on the role of bacterial membrane structures (24–27), but other regulations which may play roles are not yet identified. Recently, we have adopted gas chromatography-mass spectrometry (GC-MS)-based metabolomics to investigate the metabolic regulation of serum-resistant *Streptococcus agalactiae* in response to fish plasma. We found that *S. agalactiae* mounted the metabolic trick to cope with the complement-mediated killing, which was reverted and enhanced by exogenous malic acid and adenosine, the two crucial biomarkers identified from the serum resistance metabolome, respectively (28). These findings implied that metabolic modulation may contribute to the serum resistance in *S. agalactiae*.

To explore the possible role of metabolism in regulating serum resistance in *E. tarda*, we adopted our established metabolomic platform and investigated the characteristics of the serum resistance-associated metabolome, by which we may identify the pathways contributing to serum resistance. And we found that EIB202 promoted the TCA cycle and enhanced membrane potential as a metabolic trick against the serum complement-mediated killing. The TCA cycle positively regulates the membrane potential through generating NADH, which is used as electron carrier to increase proton motive force or membrane potential. This metabolic flow represents an unknown mechanism for serum resistance in Gram-negative bacteria. Thus, the development of inhibitors for TCA cycle or screening metabolites that attenuate TCA cycle activation could possibly facilitate the clearance of serum-resistant pathogens.

## Materials and Methods

### Serum Sample and Ethic Statement

The fish plasma was prepared from adult crucian carp, *Carassius carassius*, according to the standard protocol. The human plasma was prepared from normal human adult during annual health examination. Each individual signed the consent form that informs the use of the serum for research purpose. This study was conducted in accordance with the recommendations in the Guide for the Care and Use of Laboratory Animals of the National Institutes of Health and maintained according to the standard protocols.^1^ All experiments were approved by the Institutional Animal Care and Use Committee of Sun Yat-sen University (Animal welfare Assurance Number: 16).

#### Bacterial Strain and Culture Condition

The bacterial strain *E. tarda* EIB202 used in this study was obtained from Professor Yuanxin Zhang, East China University of Science and Technology. The complete genome sequence of EIB202 was published in 2009 (29). A single colony was propagated in tryptic soy broth (TSB) for 16 h at 30°C. The cultures were diluted into 1:100 using fresh TSB medium and grown at 30°C. Bacterial cells were harvested at 0.6 of OD_600_ by centrifugation at 8,000 *g* for 5 min at 4°C and washed three times with saline solution.

#### Sample Preparation and Percent Survival of EIB202 in Response to Complement-Mediated Killing

The bacterial samples in response to complement-mediated killing were prepared as described previously (30). Blood of crucian carps was collected *via* vein puncture and 0.02% of the heparin was used for anticoagulation. Plasma was isolated by centrifugation. Human plasma was pooled from 100 healthy human donors. Both kept −80°C for use. Bacterial pellet from 3 mL of the harvested bacteria above was collected. Then 50 and 100 µL of fish plasma or human plasma were added in the experimental groups with or without succinate or inhibitors, and an equal volume of sterile saline was added in the control group. The mixtures were cultured in 200 rpm at 30°C for 2 h. Bacterial cells were collected using centrifugation at 8,000 *g* for 10 min at 4°C and suspended in 3 mL sterile saline. The samples were serially diluted with sterile saline and 10 µL aliquots were spot plated onto TSB agar plates. The plates were cultured at 30°C for 24 h and CFU was calculated in the next day. The percent survival was determined by dividing the CFU of the treatment sample by the CFU of the sterile saline control. Meanwhile, bacteria were collected and washed twice with the same centrifugation protocol as above for preparation of the GC-MS sample.

#### Metabolomic Profiling

Sample preparation for GC-MS was performed as described previously (31). Briefly, equivalent cells were extracted with 1,000 µL of cold methanol, which contained 10 µL of 0.1 mg/mL adonitol (Sigma) as internal analytical standard. The cells were lysed by sonication for 10 min at 30% intensity and were centrifuged for 10 min at 12,000 *g* at 4°C. Then, 1,000 µL of supernatant was transferred into a new Eppendorf tube and was dried by vacuum centrifugation device (LABCONCO). Finally, the samples were performed on a GC-MS system. Each sample had four biological replicates with two technical repeats.

Gas chromatography-mass spectrometry analysis was carried out with a variation on the two-stage technique as described previously (32). Before analysis, samples were derivatized. First, carbonyl functions were protected by methoximation through a 100 min 37°C reaction with 80 µL of 20 mg/mL methoxyamine hydrochloride (Sigma) in pyridine. Then, acidic protons were exchanged against trimethylsilyl group by 37°C reaction with 80 µL of *N*-methyl-*N*-trimethylsilyltrifluoroacetamide (MSTFA, Sigma) for 30 min. The derivatized sample of 1 µL aliquot was injected into a dodecyl benzenesulfonic acid (DBS) column (30 m length × 250 µm i.d. × 0.25 µm thickness, Agilent, 5975C/7890A) using splitless model. The temperature-programmed procedure started at 85°C for 5 min and then increased to a final temperature of 330°C and was held constant for 5 min, followed by a rate of 15°C/min. Electron impact ionization (EI) mode was selected, and ionization was of 70 eV energy. Helium was used as the carrier gas with the flow rate of 1 mL/min. The range of mass full scan mode was 50–600 *m/z*.

#### Data Processing and Statistical Analysis

Metabolites from the GC-MS spectra were identified by searching in National Institute of Standards and Technology (NIST) library, using the NIST MS search 2.0. The resulting data matrix were normalized using the concentrations of added internal standards which were subsequently removed so that the data could be used for modeling consisted of extracted compound. Peak areas of all identified metabolites were normalized by ribitol as internal standard. *Z*-score and hierarchical clustering were used to analyze the normalization area. Normalized data were used for hierarchical clustering in the R platform with the package “g plots,” using the distance matrix calculated with the method of Euclidean. False discovery rates (FDR), which indicated the proportion of the true null hypotheses in the research, were determined from the *q*-value. iPath analysis was carried out by a web-based tool^2^ for the visualization and analysis of cellular pathways (33).

#### The Quantitative Real-Time PCR (qRT-PCR)

The quantitative real-time PCR was carried out as described previously (34). Total RNA of each sample was isolated with Trizol (Invitrogen, USA). The RNA was then quantified spectrophotometrically. qRT-PCR was carried out on 1 µg of total RNA by using a PrimeScript™ RT reagent kit with gDNA eraser (TAKARA, Japan) according to manufacturer’s instructions. qRT-PCR was performed in 384-well plates with a total volume of 10 µL containing 5 µL 2× SYBR Premix Ex Taq™, 2.2 µL PCR-grade water, 2 µL cDNA template, and 0.4 µL each of forward and reverse primers (10 µM).

All the primers used for qRT-PCR were shown in Tables S1 and S2 in Supplementary Material. All the samples were assayed in biological triplicate and run on CFX384 Touch (Bio-Rad, USA) according to the manufacturer’s instructions. The cycling parameters were listed as follows: 95°C for 30 s to activate the polymerase; 40 cycles of 95°C for 10 s; and 58°C for 30 s. Fluorescence measurements were performed at 72°C for 1 s during each cycle. Cycling was terminated at 95°C with a calefactive velocity of 5°C/s to obtain a melting curve. To analyze the relative expression level of target gene, we converted the data to percentages relative to the value of no treatment group.

### Generation of *sucA* and *sucB* Deletion Mutants

Knockout of *sucA* and *sucB* were performed using one-step inactivation of chromosomal genes. The primers used to amplify kanamycin cassette from pKD13 are: *sucA*-KOF: 5′-ATATTCACCACGGCGAATAACAGGCTTTACAAGCTTAAGGGATCACAATGATTCCGGGGATCCGTCGACC-3′; *sucA*-KOR: GTCGCGAATGCGGGCGACAGCGCCCGCACCCTTTATTCCACATTCAGGGCTGTAGGCTGGAGCTGCTTCG-3′; *sucB*-KOF: 5′-GGCGCTGTCGCCCGCATTCGCGACACGCATTAATACAAGGATAAACAATGATTCCGGGGATCCGTCGACC-3′; *sucB*-KOR: 5′-GCGGGCCTGTGCATAGCACGGATCACACGGAGTTACACATCCAGCAGCAGTGTAGGCTGGAGCTGCTTCG-3′. The PCR products were transformed to *E. tarda* EIB202 expressing lambda red recombinase. The transformants were selected on 50 µg/mL kanamycin. The deletion mutants were confirmed by PCR. Kanamycin cassette was removed by transforming pCP20 plasmid.

#### Measurement of Enzyme Activity

The harvested cells were added to metabolites and antibiotic and incubated at 30°C for 6 h. After incubation, cells were collected and re-suspended in sterile saline to OD_600_ = 1.0. Samples with 1 mL were collected by centrifugation at 8,000 rpm for 5 min. Pellets were re-suspended in PBS and broke down by sonication for 2 min at a 200 W power setting on ice, and then centrifuged at 12,000 rpm for 10 min to remove insoluble material. Supernatants containing 400 µg total proteins were transferred to pyruvate dehydrogenase (PDH) reaction mix (0.5 mM MTT, 1 mM MgCl_2_, 6.5 mM PMS, 0.2 mM TPP, 2 mM sodium pyruvate, 50 mM PBS), ketoglutarate dehydrogenase (KGDH) reaction mix (0.5 mM MTT, 1 mM MgCl_2_, 6.5 mM PMS, 0.2 mM TPP, 50 mM alpha-ketoglutaric acid potassium salt, 50 mM PBS), or succinate dehydrogenase (SDH) reaction mix (0.5 mM MTT, 13 mM PMS, 5 mM sodium succinate, 50 mM PBS), to a final volume of 200 µL in 96-well plate. Subsequently, the plate was incubated at 37°C for 5 min for SDH/PDH/OGD assays, and then measured at 566 nm for colorimetric readings. The plate was protected from light during the incubation. Experiments were repeated at least in three independent biological replicates.

#### Measurement of Bacterial Membrane Potential

Measurement of membrane potential was described previously (35). *E. tarda* EIB202 cells were adjusted to 10^7^ CFU/mL in saline. The 10^7^ CFU/mL diluted cells were strained with 10 µM DiOC2(3) for 30 min at 37°C. Aliquots 1 mL of culture was added into flow tubes before analysis. Samples were analyzed on a FACSCalibur flow cytometer (Becton Dickinson, San Jose, CA, USA). Flow cytometry analyses were done at 37°C. Each sample was observed with forward versus side scatter and gated before the acquisition of data. Settings were optimized according to the manual. DIOC2(3)’s green fluorescence (488 nm excitation, 530 nm emission) is cell size-dependent and membrane potential-independent. The size and membrane potential determined the intensity of Red (488 nm excitation, 610 nm emission) fluorescence. The diverse ratios of red and green indicated fluorescence intensity values of the gated populations. Computational formula of membrane potential: $\text{Log}\left(10^{3/2}\times \left(\frac{\text{red fluorescence}}{\text{green fluorescence}}\right)\right)$. Experiments were repeated at least in three independent biological replicates.

#### Relative Fluorescence Intensity Detection of C9 Neoantigen on the Bacterial Outer Membrane

10^6^ CFU/mL diluted cells were mixed with 0.5 µg C9 neoantigen monoclonal antibody (Hycult Biotech Inc., Netherland) for 0.5 h at 37°C. Aliquots 1 mL of culture was added into flow tubes and then analyzed on a FACSCalibur flow cytometer. Experiments were repeated at least in three independent biological replicates.

## Results

### EIB202 Is Intrinsically Resistant to Complement-Mediated Killing

**Figure 1:**
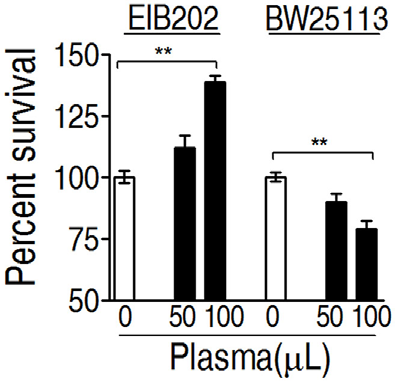
Percent survival of *Edwardsiella tarda* EIB202 and *Escherichia coli* K12 BW25113 cells in the presence and absence of crucial carp plasma. Results are displayed as mean ± SEM, and significant differences are identified (**p < 0.01) as determined by Student’s *t*-test. At least three biological repeats were carried out.

To investigate whether *E. tarda* EIB202 is resistant to serum complement-mediated killing, percent survival of EIB202 was detected in the presence or absence of crucian carp plasma, where *Escherichia coli* K12 was treated with the same amount of plasma as control. EIB202 grew faster in crucian carp plasma, whereas *E. coli* was killed in the same plasma (Figure 1). These results indicate that EIB202 is serum-resistant.

### Metabolomic Profile of EIB202 Exposed to Crucian Carp Plasma

To identify the metabolic profile that was required for EIB202 survived from crucian carp plasma, GC-MS-based metabolomics was adopted to investigate effect of crucian carp plasma on the metabolic profile of EIB202. To perform functional metabolomics analysis, four biological replicates for each group, two technical replicas for each biological replica were included, yielding a total of 16 datasets. A total of 166 aligned individual peaks were obtained from each bacterial sample after the removal of internal standards and the known peaks for solvent, leading to the identification of 63 metabolites. The abundance of the identified metabolites including plasma-treated group and the control group was listed in Figure S1A in Supplementary Material. Pearson correlation coefficient between two technical replicates varied between 0.994 and 0.999 (Figure S1B in Supplementary Material), ensuring the confidence of the dataset for further analysis. These metabolites were classified into five categories: carbohydrates (33.3%), amino acids (31.7%), lipids (20.6%), nucleotides (9.5%), and others (4.8%) (Figure S1C in Supplementary Material). These results indicate that *E. tarda* adopted metabolic shift when exposed to crucian carp plasma.

### Metabolomic Profiling Variations of EIB202 Exposed to Crucian Carp Plasma

**Figure 2:**
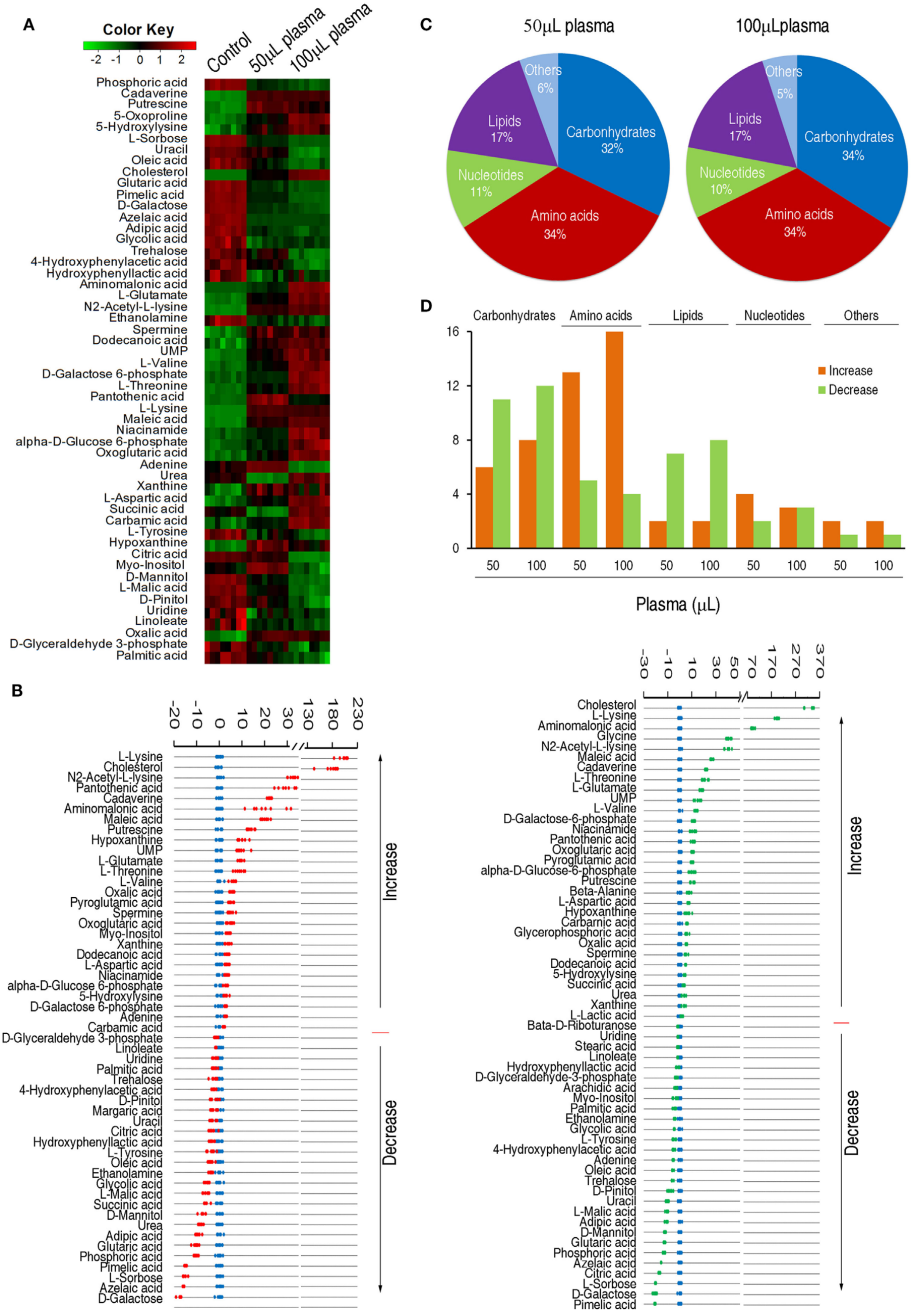
Differential abundance of metabolites in response to serum complement-mediated killing. **(A)** Heat map of differential abundance of metabolites (row). Green and red indicate decrease and increase of the metabolites scaled to mean and standard deviation of row metabolite level, respectively (see color scale). **(B)** *Z*-score plots of differential abundances of metabolites based on control. Data from the two tested groups are separately scaled to the mean and standard deviation of control. Each point represents one metabolite in one technical repeat and colored by sample types (blue: control, red: 50 µL plasma group, green: 100 µL plasma group). **(C)** Percentage of differential abundance of metabolites in five categories. **(D)** Number of metabolites increased and decreased in the five categories.

To identify the variations of the metabolomic profile, Chi-square test was used to detect metabolites of differential abundance. 53 and 59 differential metabolites were identified in 50 and 100 µL plasma-treated groups as to control group, respectively. To better visualize this relationship, hierarchical clustering was used to arrange the metabolites on the basis of their relative levels across samples (Figure 2A). *Z*-score plots, displaying the levels of differential metabolites, were generated to compare the experimental groups and the control group. Among the differential metabolites, 27 metabolites were up-regulated and 26 metabolites were down-regulated in the 50 µL plasma-treated group, whereas 31 metabolites were up-regulated and 28 metabolites were down-regulated in the 100 µL plasma-treated group (Figure 2B). The metabolites of differential abundance were classified into five categories ranking from high to low: amino acids (34%) > carbohydrate (32%) > lipids (17%) > nucleotides (11%), and others (6%) in the 50 µL plasma-treated group, and amino acids (34%) = carbohydrate (34%) > lipids (17%) > nucleotides (10%), and others (5%) in the 100 µL plasma-treated group (Figure 2C). Among these metabolites, the number of the increased and decreased metabolites was listed in Figure 2D. This result showed that the change of abundance of the metabolites was associated with the dose of plasma, typically enriched in carbohydrates, amino acids, and lipids. These results indicate that EIB202 mounts a differential metabolome in serum.

### Enrichment of Metabolic Pathways Contributing to Serum Resistance

**Figure 3:**
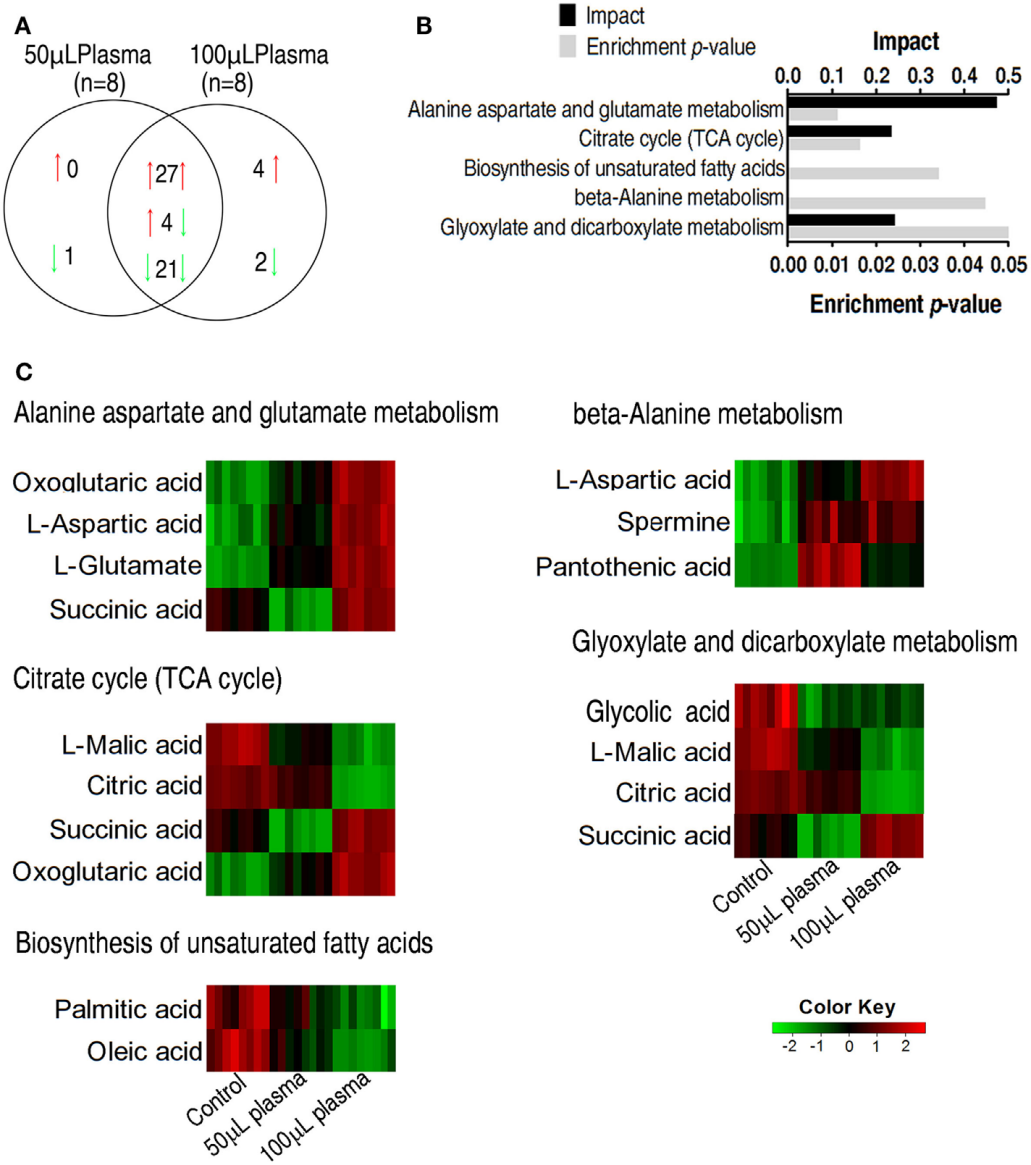
Enriched pathways and their metabolites in response to serum complement-mediated killing. **(A)** The distribution of differential abundance of metabolites in the two plasma-treated groups, showing overlapping and unique metabolites. Red and green arrows indicate increase and decrease, respectively. **(B)** Enriched pathways by 52 overlapping metabolites between the two plasma-treated groups. **(C)** Heat map of differential abundance of metabolites in the enriched pathways. Red and green, respectively, indicate increase and decrease of the metabolites scaled to mean and standard deviation of row metabolite level of control (see color scale).

The two plasma-treated groups had metabolites in common that shared 27 increased metabolites and 21 decreased metabolites in addition of 4 metabolites with reversed change (Figure 3A). Pathways were enriched in the 52 metabolites, leading to the identification of five pathways, including alanine, aspartate, and glutamate metabolism, the TCA cycle, biosynthesis of unsaturated fatty acids, beta-alanine metabolism, glyoxylate, and dicarboxylate (Figure 3B). Exposure of EIB202 to the plasma increased abundance of metabolites in three pathways (alanine, aspartate, and glutamate metabolism, beta-alanine metabolism and glyoxylate, and dicarboxylate metabolism) but the TCA cycle contained both increased and decreased abundance of metabolites (Figure 3C). On the other hand, a total of 11 metabolites were involved in the 5 pathways, where succinate belongs to 3 pathways, and oxoglutaric acid, asparatic acid, malic acid, and citric acid belongs to 2 pathways, and the other 6 metabolites belongs to only 1 pathway. Of notice, the four metabolites play a role in the TCA cycle (Figure 3C). These results indicate that regulation of metabolic pathways contributes to serum resistance of EIB202, in which the TCA cycle might be involved.

### The Enhanced TCA Cycle As a Characteristic Feature of the Serum Killing

**Figure 4:**
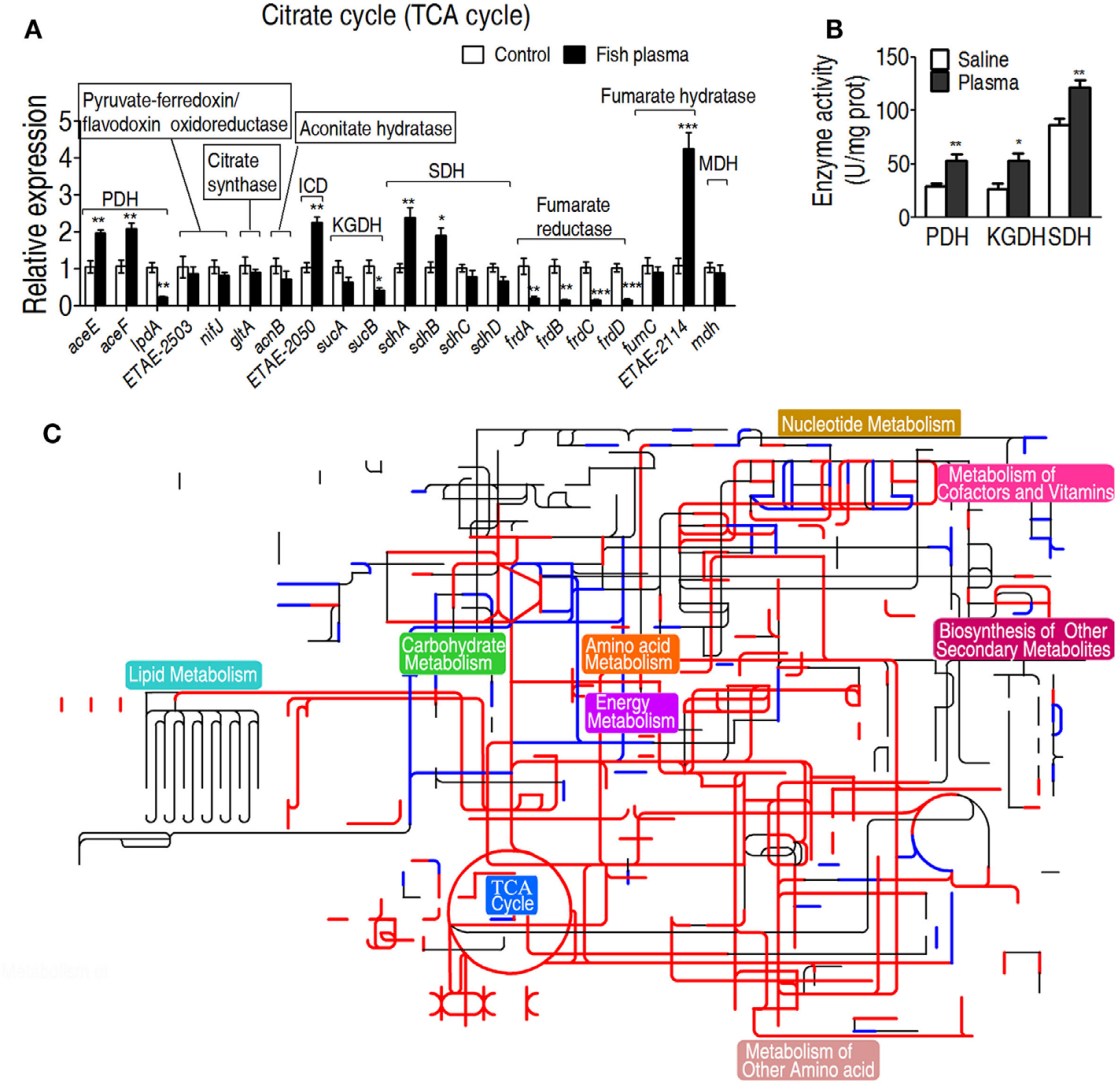
Characteristics of the tricarboxylic acid (TCA) cycle in response to serum killing. **(A)** Quantitative real-time PCR (qRT-PCR) for gene expression of the TCA cycle and alanine, aspartate, and glutamate metabolism. **(B)** Activity of pyruvate dehydrogenase (PDH), ketoglutarate dehydrogenase (KGDH), and succinate dehydrogenase (SDH) in the presence or absence of crucian carp plasma. **(C)** Interactive Pathways Explorer (iPath) analysis. Metabolic network pathways in *Edwardsiella tarda* EIB202 are analyzed with iPath 2.0 (http://pathways.embl.de/iPath2.cgi). Analyses of the 46 differential metabolites (the others are not recognized in the metabolic network pathways), 6 increased and 6 decreased gene expressions and elevated PDH, KGDH, and SDH activity resulting from *E. tarda* EIB202 in response to crucian carp plasma provide a better insight into the effects. Red = increase, green = decrease. Results are displayed as mean ± SEM, and significant differences are identified (*p < 0.05; **p < 0.01; ***p < 0.001) as determined by Student’s *t*-test. At least three biological repeats were carried out.

To further investigate the relationship between the TCA cycle and serum resistance, qRT-PCR was used to detect expression of genes in the TCA cycle and PDH (it provides acetyl-CoA to fuel the TCA cycle), where 19 and 3 genes were detected, respectively. Among the examined genes, the expression of six genes was elevated, of nine genes was unchanged and of six genes was decreased. Among the decreased genes, *frdA/B/C/D* encode fumarate reductase that reduces fumarate to succinate, which supports the increased TCA cycle; *lpd* encoding a subunit of PDH was coupled with the other two elevated *aceE* and *aceF* of PDH; *sucA* and *sucB*, encoding alpha-KGDH, were unaffected (Figure 4A). These results further confirmed that the gene expression of PDH and the TCA cycle was increased in response to crucian carp plasma. Then, activities of PDH, KGDH, and SDH were measured. PDH catalyzes the irreversible oxidative decarboxylation of pyruvate to acetyl-CoA. KGDH catalyzes the conversion of alpha-ketoglutarate to succinyl-CoA and produces NADH, directly providing electrons for the respiratory chain. SDH catalyzes succinate oxidation in the TCA cycle and transfers the electrons to quinones in the membrane, thus constituting a part of the aerobic respiratory chain (known as complex II). The activities of the three enzymes were increased in EIB202 when exposed to plasma (Figure 4B). Interactive Pathways Explorer (iPath) constructs metabolic pathways that give an overview of the complete metabolism in biological systems. With iPath, a comparative metabolic pathway analysis on differential metabolites, genes and enzymes between the experiment group and control was directly visualized, where red and green lines represent increase and decrease in treated group, respectively (Figure 4C). The plasma treatment led to the elevation of most metabolic pathways, in which TCA cycle plays a crucial role, because the increased TCA cycle can result in elevation of most metabolic pathways. These results support the conclusion on the elevated response of alanine, aspartate, and glutamate metabolism, the TCA cycle, and pyruvate metabolism to the complement-mediated killing. Since alanine, aspartate, and glutamate metabolism, and pyruvate metabolism fuel the TCA cycle, further investigation focuses on the TCA cycle.

### The Increased TCA Cycle As a Result of Serum Resistance

**Figure 5:**
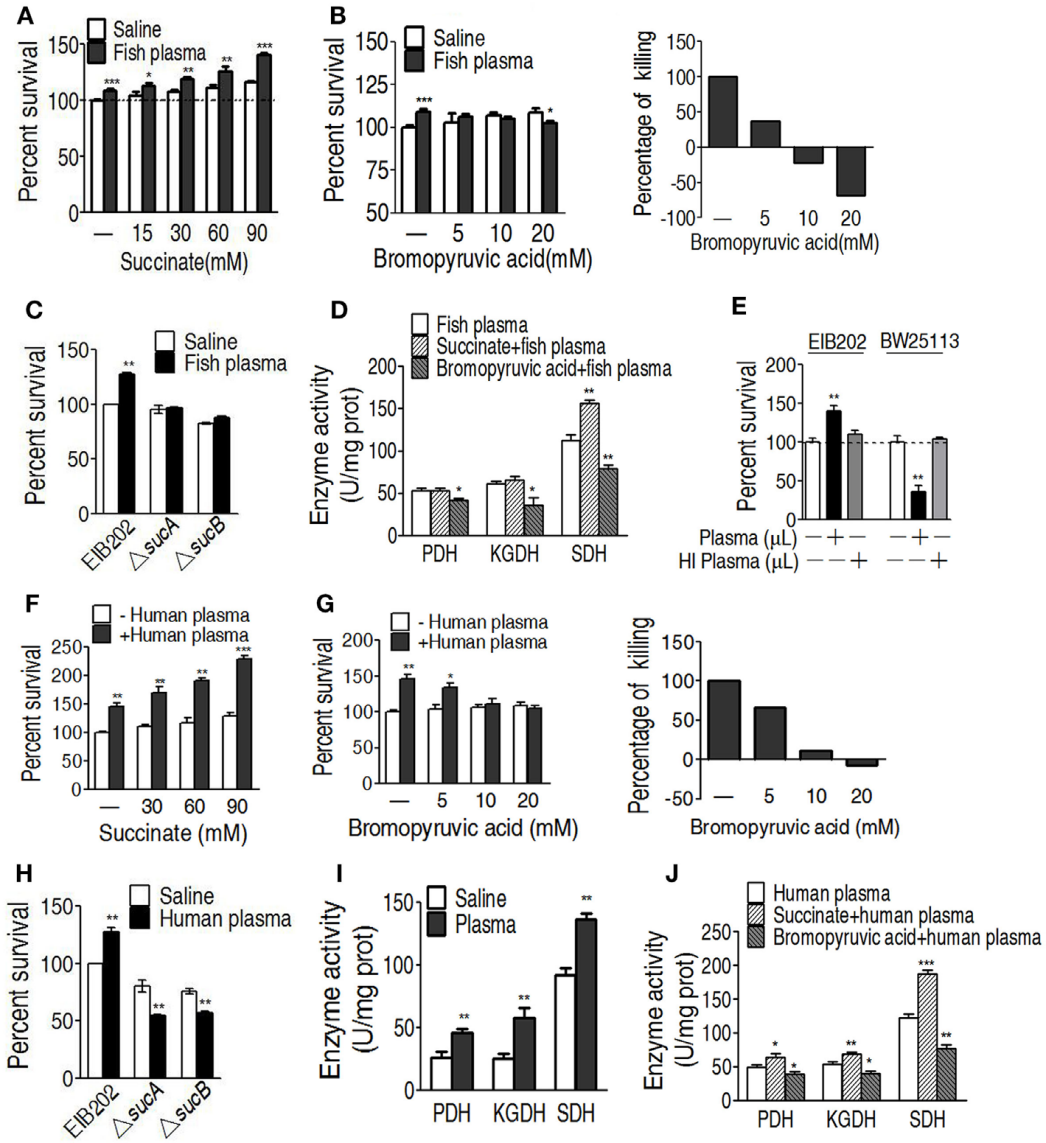
Effect of regulating the tricarboxylic acid (TCA) cycle on the serum killing. **(A)** Percent survival of EIB202 in the presence of succinate plus carp plasma. **(B)** Percent survival of EIB202 in the presence of the indicated bromopyruvate plus carp plasma (left). For relative percentage of killing, the elevated percent survival in the sample without crucial carp plasma treatment is taken 100% as a standard (first column); the difference with and without plasma plus the indicated bromopyruvate was compared with the standard (right). **(C)** Activity of pyruvate dehydrogenase (PDH), ketoglutarate dehydrogenase (KGDH), and succinate dehydrogenase (SDH) in the presence of succinate or bromopyruvate plus carp plasma. **(D)** Percent survival of EIB202 in the presence or absence of *sucA* or *sucB* plus crucial carp plasma. **(E)** Percent survival of *Edwardsiella tarda* EIB202 and *E. coli* K12 BW25113 cells in the presence and absence of human plasma or heat-inactivated (HI) human plasma. **(F)** Percent survival of EIB202 in the presence of the indicated succinate plus human serum. **(G)** Percent survival of EIB202 in the presence of the indicated bromopyruvate plus human serum. For relative percentage of killing, the elevated percent survival in the sample without human plasma treatment is taken 100% as a standard (first column); the difference with and without plasma plus the indicated bromopyruvate was compared with the standard (right). **(H)** Percent survival of EIB202 in the presence or absence of *sucA* or *sucB* plus human plasma. **(I)** Enzyme activity of PDH, KGDH and SDH in EIB202 when exposed to saline of human plasma. **(J)** Enzyme activity of PDH, KGDH and SDH in the presence or absence of succinate or bromopyruvate plus human serum. Results are displayed as mean ± SEM, and significant differences are identified (*p < 0.05, **p < 0.01, ***p < 0.001) as determined by Student’s *t*-test. At least three biological repeats were carried out.

To further demonstrate the role of the increased TCA cycle in serum resistance, exogenous succinate was used to fuel the TCA cycle. The enhanced TCA cycle elevated the survival of EIB202 exposed to crucian carp plasma in a succinate dose-dependent manner (Figure 5A). On the contrary, PDH inhibitor bromopyruvate reduced the survival in a manner of bromopyruvate dose (Figure 5B). When *sucA* or *sucB*, encoding KGDH, was absent, lower percent survival of EIB202 was detected in the presence of crucian carp plasma than in the absence of the plasma (Figure 5C). Correspondingly, the exogenous succinate and bromopyruvate increased and decreased activity of the three enzymes except for KGDH, respectively (Figure 5D). *E. tarda* also causes human diseases (4, 5). Thus, the crucian carp plasma was replaced with human plasma to test whether human plasma led to similar outputs under the two treatments. As expected, EIB202 was resistant to human plasma, but *E. coli* K12 BW25113 was not (Figure 5E). Exogenous succinate and bromopyruvate, respectively, promoted and inhibited the survival of EIB202 in response to the human plasma-mediated killing in a dose-dependent manner (Figures 5F,G). *sucA* or *sucB* deletion mutants had lower percent survival in the presence of human plasma (Figure 5H). Activities of PDH, KGDH, and SDH were increased in EIB202 when exposed to human plasma (Figure 5I). Exogenous succinate and bromopyruvate increased and decreased the activity of the three enzymes, respectively (Figure 5J). These results indicate that the TCA cycle is crucial to the serum resistance of EIB202 in response to human plasma- and crucian carp plasma-mediated killing.

### The Increased Membrane Potential As a Result of Serum Resistance

**Figure 6:**
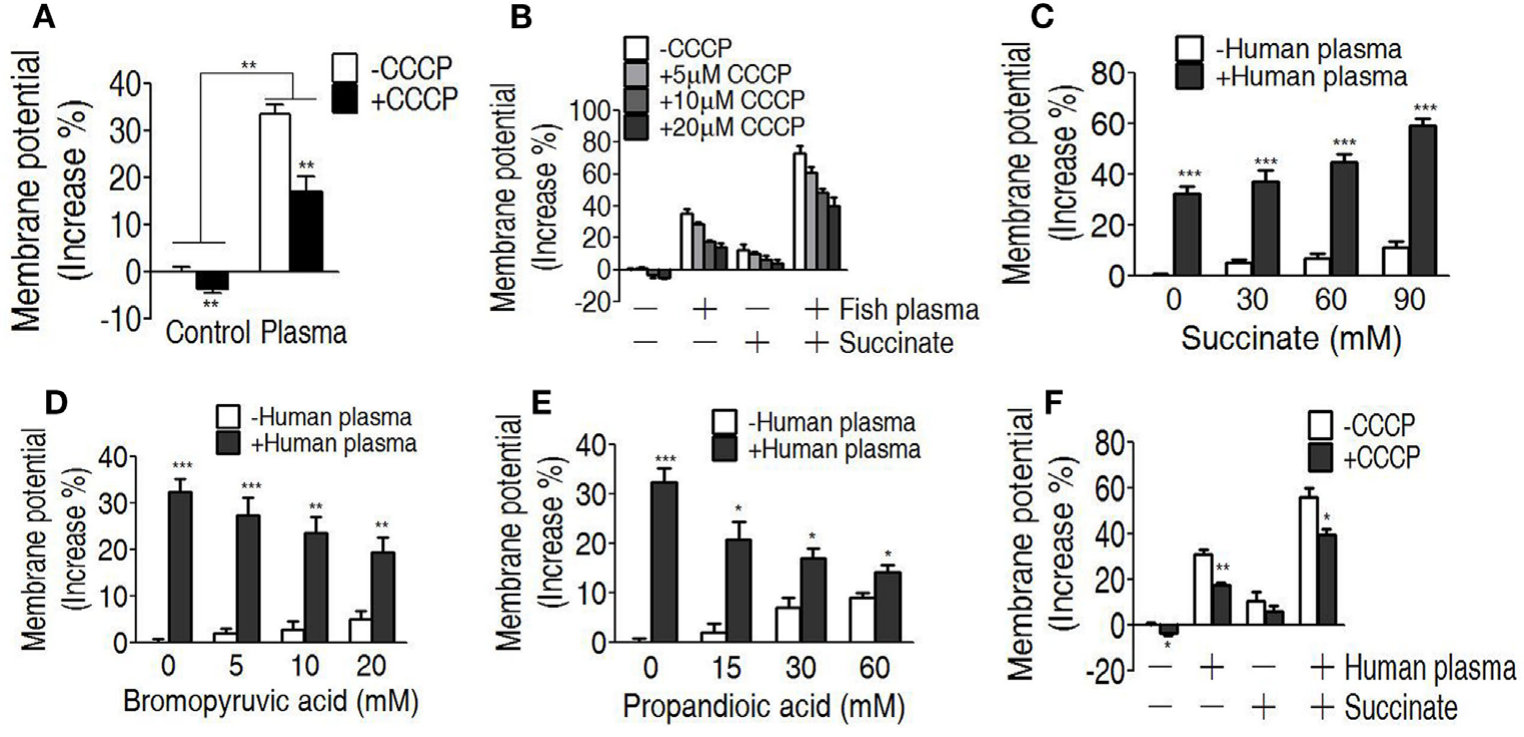
Measurement of membrane potential in the serum killing. **(A)** Membrane potential of EIB202 in the presence or absence of crucial carp plasma plus carbonyl cyanide-m-chlorophenylhydrazone (CCCP). **(B)** Membrane potential of EIB202 in the presence or absence of succinate and crucial carp plasma plus the indicated CCCP. **(C)** Membrane potential of EIB202 in the presence or absence of the indicated succinate plus human plasma. **(D)** Membrane potential of EIB202 in the presence or absence of the indicated bromopyruvate plus human plasma. **(E)** Membrane potential of EIB202 in the presence or absence of the indicated propandioic acid plus human plasma. **(F)** Membrane potential of EIB202 in the presence or absence of succinate or human serum plus CCCP. Results are displayed as mean ± SEM, and significant differences are identified (*p < 0.05, **p < 0.01, ***p < 0.001) as determined by Student’s *t*-test. At least three biological repeats were carried out.

We reasoned that the elevated TCA cycle promotes NADH generation, thereby leading to the elevated membrane potential. To demonstrate this, membrane potential of EIB202 was detected in the presence or absence of succinate or bromopyruvate plus crucian carp plasma or human plasma. Meanwhile, carbonyl cyanide-m-chlorophenylhydrazone (CCCP), an inhibitor of membrane potential, was used to confirm these results. Higher membrane potential was detected in EIB202 exposed to crucian carp plasma than the one not, which was partly inhibited by CCCP (Figure 6A). Synergy of crucian carp plasma with succinate led to higher membrane potential than plasma or succinate alone, which was also inhibited by CCCP in a dose-dependent manner (Figure 6B). When crucian carp plasma was replaced with human plasma, succinate promoted membrane potential in a dose-dependent manner (Figure 6C). On the contrary, bromopyruvate and SDH inhibitor, propandioic acid, reduced the membrane potential with the increased doses (Figures 6D,E). CCCP inhibited the membrane potential caused by human plasma and plus succinate (Figure 6F). These results indicate that bacterial membrane potential is related to serum resistance.

### Effect of Membrane Potential on Binding with Complement MAC

**Figure 7:**
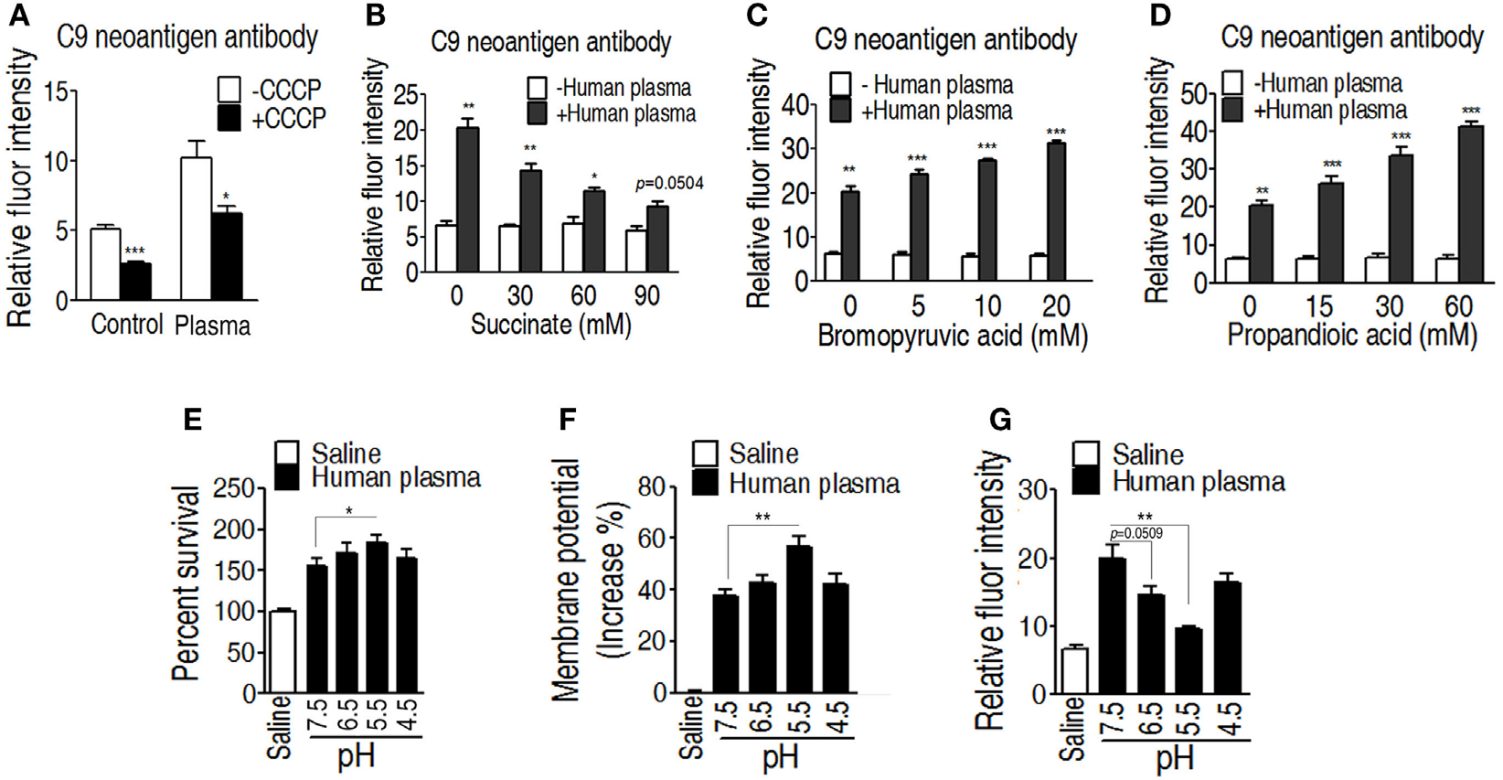
Measurement of C9 neoantigen in the serum killing. **(A)** Neoantigen of EIB202 in the presence or absence of human plasma. **(B)** Neoantigen of EIB202 in the presence or absence of human plasma plus the indicated succinate. **(C)** Neoantigen of EIB202 in the presence or absence of human plasma plus the indicated bromopyruvate. **(D)** Neoantigen of EIB202 in the presence or absence of human plasma plus the indicated propandioic acid. **(E–G)** Percent survival **(E)**, membrane potential **(F)**, and neoantigen **(G)** of EIB202 in the human plasma buffered the indicated pH. Results are displayed as mean ± SEM, and significant differences are identified (*p < 0.05, **p < 0.01, ***p < 0.001) as determined by Student’s *t*-test. At least three biological repeats were carried out.

One of the mechanisms of serum resistance is attributed to the reduced deposition of MAC that forms channel pore on bacterial membrane, causing cell lysis. Thus, we reasoned that the decreased binding MAC to the membrane may be determined by the level of membrane potential that is associated with the TCA cycle. To test this hypothesis, a neoantigen of EIB202 surface was measured in the presence or absence of exogenous succinate or inhibitor bromopyruvate plus human serum or/and CCCP. The neoantigen is a neoepitope on the 61-kDa complement component C9, an integrated component in the MAC. Higher level of neoantigen was detected in EIB202 with human plasma than without. CCCP reduced the neoantigen binding to EIB202 in regardless of the presence of plasma (Figure 7A). Furthermore, exogenous succinate reduced the neoantigen binding, while brompyruvate or propandioic acid increased the binding (Figures 7B–D). The membrane potential is related to environmental pH (28). Percent survival of EIB202 was detected in the human plasma with different pH. The highest survival of EIB202 was found at pH 5.5, which is similar to the isoelectric point (PI) of complement C3. When pH was increased or decreased, the percent survival was reduced (Figure 7E). Accordingly, the corresponding membrane potential was detected (Figure 7F), whereas the inverse neoantigen was found in EIB202 exposed to the same human serum with different pH (Figure 7G). These results indicate that bacterial membrane potential is a crucial factor to the binding with complement and the form of the MAC.

## Discussion

Bacterial pathogens with serum resistance pose a big challenge to human health and animal breeding. They can escape from serum complement-mediated killing that is a rapid and potent measure of innate immunity against bacteria (36, 37). So far, three mechanisms have been reported for serum resistance in bacteria: cleavage of complement components with protease; inhibition of complement activation through recruitment of factors such as factor H and C4BP to the bacterial cell surface; and lipopolysaccharide- and capsular polysaccharide-mediated suppression of complement activation (38, 39), all of which leads to the failure of MAC formation. Since the three pathways are associated with membrane structures, almost all of the studies investigate the physical nature of outer membrane, and the functions of outer membrane proteins in serum resistance in Gram-negative bacteria (40, 41). This study, however, aims to explore a metabolic response of EIB202 to serum complement-mediated killing, by which the identified metabolic strategies can be used to counter against the complement-mediated killing. Our results showed that EIB202 exhibits differential metabolome when they encounter with crucian carp plasma, which is consistent with our previous report on the metabolic regulation of *S. agalactiae* to yellow group plasma (28). These results indicate that the metabolic regulation is a strategy in bacteria to cope with the serum complement-mediated killing. Thus, serum resistance may be viewed as a metabolic shift, and could be a whole cell response to the external stress.

Modulating metabolome could repurpose for overcoming bacterial resistance to antibiotics, or host’s response to bacterial infections through identifying the key metabolic pathways and crucial biomarkers (35, 42–45). The metabolome of EIB202 exposed to crucian carp plasma was characterized with enhanced alanine, aspartate, and glutamate metabolism, TCA cycle, and the elevated succinate as the most crucial biomarker. Further investigation on gene expression, enzyme activity, and metabolic pathways showed the two enhanced pathways and PDH in response to crucian carp plasma. The enhanced alanine, aspartate, and glutamate metabolism, and pyruvate metabolism may fuel the TCA cycle. Thus, the TCA cycle was carefully examined when EIB202, a strain that infects fish, mouse, and human being demonstrated previously (42), was exposed to human plasma or crucian carp plasma. Both human plasma and crucian carp plasma have been tested due to the broad host range of *E. tarda* (1–3). Interestingly, the action of human plasma and fish plasma on EIB202 has slight difference, where EIB202 grew a bit faster in human plasma than in fish plasma when incubated in the same amount of plasma (Figures 1 and 5E). This may be due to the different components between these two types of plasma that resulted in different response of EIB202 to the complement-mediated killing. This possibility could be partly confirmed with the results that EIB202 grew faster in fish plasma than in human plasma when succinate was supplemented (Figures 5A,F). In addition, commercial antibody to a neoantigen of crucian carp is not available, which makes subsequent investigation of the serum resistance impossible. Our results showed that the promotion of the TCA cycle by exogenous succinate and the inhibition of PDH by bromopyruvate and of SDH by propanedioic acid lead to the increased and decreased survival of EIB202, respectively. Actually, the importance of the TCA cycle in the pathogenesis of *Edwardsiella* species has been observed by others. The deletion or mutation of the genes of TCA cycle significantly attenuate the pathogenesis of *E. ictaluri* (46, 47). In addition, the TCA deletion mutants are potent vaccine candidates (48). But how the TCA cycle contributes to bacterial pathogenesis besides serum resistance requires further investigation. These results support the conclusion that the elevated TCA cycle contributes to the serum resistance in EIB202, and thereby the pathogen can still grow in serum.

To explore the mechanism of TCA cycle promotion increases serum resistance, we hypothesized that the TCA cycle generate more NADH, thereby increases membrane potential, which reduces the binding with the complement. Thus, the membrane potential mediating serum resistance was investigated. The demonstration that the high membrane potential is related to the high survival of EIB202 suggests that the membrane potential is essential for EIB202 to evade complement-mediated killing. The finding that the high membrane potential contributes to the high detection of the neoantigen on bacterial outer membrane indicates that the MAC binding to the outer membrane is regulated by the membrane potential. Although previous reports revealed the inter-relationship between bacterial serum resistance and membrane structures (40, 41), the membrane potential involved in the serum resistance was previously unknown. Of notice, we only investigated the key part of MAC, the C9 neoantigen, activation in the current study. The identification of the complement pathway that leads to C9 activation requires further study.

Besides the TCA cycle, we also enriched other pathways that may also impact serum resistance, including alanine, aspartate, and glutamine metabolism, biosynthesis of unsaturated fatty acids and glyoxylate and dicarboxylate metabolism. There is no direct evidence that these three pathways are involved in serum resistance. The role of unsaturated fatty acids in virulence has been documented in *Streptococcus mutans*, where the loss of *fabM* gene, the only gene for unsaturated fatty acid biosynthesis, make the bacteria avirulent and poorly transmissible from host to host (49). The alanine, aspartate, and glutamine are amino acid metabolism that plays roles in utilizing nitrogen sources to produce energy, which may be essential for bacterial pathogenesis inside the host (50). While the glyoxylate and dicarboxylate metabolism is poorly explored in bacteria except that in *Mycobacterium tuberculosis*, which is essential for their growth inside macrophage as well as the virulence (51). Thus, this pathway may involve in serum resistance by connecting with other pathways like purine metabolism, glycine, serine, and threonine metabolism, and ascorbate and aldarate metabolism (51, 52). Actually, all of the three pathways are interconnected with each other through the TCA cycle by providing fuels including oxaloacetate and acetyl-coenzyme A. This is the reason that we only focused on the TCA cycle in this study. But it is also worthy to investigate how the other three pathways contribute to serum resistance in a TCA-independent manner.

**Figure 8:**
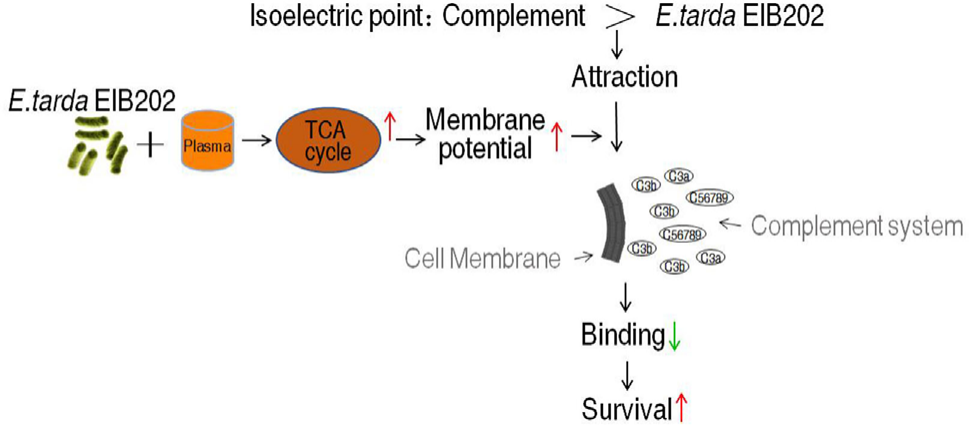
Proposed mechanism for EIB202 escaping from serum complement-mediated killing.

Our current finding suggests a membrane potential-dependent mechanism of serum resistance. A model of this overcoming strategy was summarized in Figure 8. Briefly, the isoelectric point (pI) of most components of complement caspase is around 5.5 (for example, C3 and C4) (53), and Gram-negative bacteria normally has lower pI (less than pH 4.0) (54). The binding between complement components and bacteria was thus dependent on membrane potential, which determines the pI difference. The less of difference, the less attraction between the bacteria and the complement. The elevated TCA cycle promoted the membrane potential when exposed to serum, which brings the pI difference even smaller, thereby less MAC is formed on the outer membrane becoming resistant to the serum. Because the membrane potential is affected by environmental pH (42), the neoantigen of EIB202 is related to the local envrionment pH, showing that the highest survival and lowest neoantigen were detected at pH 5.5, which is similar to the pI of complement C3.

In summary, GC-MS-based metabolomics was used to characterize a metabolic trick of *E. tarda* in resisting to complement-mediated killing, and to identify a serum resistance metabolome that characterized with the elevated TCA cycle. Further study showed that the elevated TCA cycle increased membrane potential. The high membrane potential reduced the binding of complement to bacterial membrane, leading to serum resistance. These findings revealed a previously unknown membrane potential-dependent mechanism by which bacteria are resistant to the complement-mediated killing.

## Ethics Statement

Serum sample and ethic statement: The fish plasma was prepared from adult crucian carp, *Carassius carassius*, according to the standard protocol. The human plasma was prepared from normal human adult during annual health examination. Each individual signed the consent form that informs the use of the serum for research purpose. This study was conducted in accordance with the recommendations in the Guide for the Care and Use of Laboratory Animals of the National Institutes of Health and maintained according to the standard protocols (http://ZFIN.org). All experiments were approved by the Institutional Animal Care and Use Committee of Sun Yat-sen University (Animal welfare Assurance Number: 16).

## Author Contributions

BP conceptualized and designed the project. Z-xC, Q-yG, ZW, JL, JW, and X-pL performed experiments. Q-yG, M-jY, and Z-gC performed data analysis. BP, Z-xC, and Z-gC interpreted the data. BP wrote the manuscript. All the authors reviewed the manuscript.

## Conflict of Interest Statement

The authors declare that the research was conducted in the absence of any commercial or financial relationships that could be construed as a potential conflict of interest.

## Footnotes

^1^http://ZFIN.org.

^2^http://pathways.embl.de.

## References

[1] Leung KY Siame BA Tenkink BJ Noort RJ Mok YK *Edwardsiella tarda* virulence mechanisms of an emerging gastroenteritis pathogen. Microbes Infect (2012) 14:26e34 10.1016/j.micinf.2011.08.005 21924375

[2] Song M Xie J Peng X Li H. Identification of protective immunogens from extracellular secretome of *Edwardsiella tarda*. Fish Shellfish Immunol (2013) 35:1932–6. 10.1016/j.fsi.2013.09.033 24099803

[3] Peng B Wang C Li H Su YB Ye JZ Yang MJ Outer membrane proteins form specific patterns in antibiotic-resistant *Edwardsiella tarda*. Front Microbiol (2017) 8:69. 10.3389/fmicb.2017.00069 28210241 PMC5288343

[4] Suezawa C Yasuda M Negayama K Kameyama T Hirauchi M Nakai T Identification of genes associated with the penetration activity of the human type of *Edwardsiella tarda* EdwGII through human colon epithelial cell monolayers. Microb Pathog (2016) 95:148–56. 10.1016/j.micpath.2016.04.007 27057670

[5] Crosby SN Snoddy MC Atkinson CT Lee DH Weikert DR. Upper extremity myonecrosis caused by *Edwardsiella tarda* resulting in transhumeral amputation: case report. J Hand Surg Am (2013) 38:129–32. 10.1016/j.jhsa.2012.10.009 23200948

[6] Katharios P Kokkari C Dourala N Smyrli M. First report of edwardsiellosis in cage-cultured sharpsnout sea bream, *Diplodus puntazzo* from the Mediterranean. BMC Vet Res (2015) 11:155. 10.1186/s12917-015-0482-x 26193880 PMC4508803

[7] Wang JJ Sun L. *Edwardsiella tarda*-regulated proteins in Japanese flounder (*Paralichthys olivaceus*): identification and evaluation of antibacterial potentials. J Proteomics (2015) 124:1–10. 10.1016/j.jprot.2015.04.011 25896741

[8] Xu TT Zhang XH *Edwardsiella tarda*: an intriguing problem in aquaculture. Aquaculture (2014) 431:129–35. 10.1016/j.aquaculture.2013.12.001

[9] Loch TP Hawke J Reichley S Faisal M Del Piero F Griffin MJ Outbreaks of edwardsiellosis caused by *Edwardsiella piscicida* and *Edwardsiella tarda* in farmed barramundi (*Lates calcarifer*). Aquaculture (2017) 481:202–10. 10.1016/j.aquaculture.2017.09.005

[10] Thune RL Fernandez DJ Benoit JL Kelly-Smith M Rogge ML Booth NJ Signature-tagged mutagenesis of *Edwardsiella ictaluri* identified virulence-related genes, including a *Salmonella* pathogenicity island 2 class of type III secretion system. Appl Environ Microbiol (2007) 73:7934–46. 10.1128/AEM.01115-07 17965213 PMC2168160

[11] Zheng J Leung KY. Dissection of a type VI secretion system in *Edwardsiella tarda*. Mol Microbiol (2007) 66:1192–206. 10.1111/j.1365-2958.2007.05993.x 17986187

[12] Dubytska LP Rogge ML Thune RL. Identification and characterization of putative translocated effector proteins of the *Edwardsiella ictaluri* type III secretion system. mSphere (2016) 1(3):e39–16. 10.1128/mSphere.00039-16 27303737 PMC4888880

[13] Rogge ML Thune RL. Regulation of the *Edwardsiella ictaluri* type III secretion system by pH and phosphate concentration through EsrA, EsrB, and EsrC. Appl Environ Microbiol (2011) 77:4293–302. 10.1128/AEM.00195-11 21551284 PMC3127708

[14] Baumgartner WA Dubytska L Rogge ML Mottram PJ Thune RL. Modulation of vacuolar pH is required for replication of *Edwardsiella ictaluri* in channel catfish macrophages. Infect Immun (2014) 82:2329–36. 10.1128/IAI.01616-13 24664505 PMC4019175

[15] Booth NJ Beekman JB Thune RL. *Edwardsiella ictaluri* encodes an acid-activated urease that is required for intracellular replication in channel catfish (*Ictalurus punctatus*) macrophages. Appl Environ Microbiol (2009) 75:6712–20. 10.1128/AEM.01670-09 19749068 PMC2772447

[16] Chen H Yang DH Han F Tan J Zhang L Xiao J The bacterial T6SS effector EvpP prevents NLRP3 inflammasome activation by inhibiting the Ca^2+^-dependent MAPK-Jnk pathway. Cell Host Microbe (2017) 21:47–58. 10.1016/j.chom.2016.12.004 28081443

[17] Crémet L Broquet A Jacqueline C Chaillou C Asehnoune K Corvec S Innate immune evasion of *Escherichia coli* clinical strains from orthopedic implant infections. Eur J Clin Microbiol Infect Dis (2016) 35:993–9. 10.1007/s10096-016-2628-6 27039343

[18] van der Maten E Westra D van Selm S Langereis JD Bootsma HJ van Opzeeland FJ Complement factor H serum levels determine resistance to pneumococcal invasive disease. J Infect Dis (2016) 213:1820–7. 10.1093/infdis/jiw029 26802141

[19] Kenedy MR Akins DR. The OspE-related proteins inhibit complement deposition and enhance serum resistance of *Borrelia burgdorferi*, the Lyme disease spirochete. Infect Immun (2011) 79:1451–7. 10.1128/IAI.01274-10 21282413 PMC3067540

[20] Nonaka M. Evolution of the complement system. Subcell Biochem (2014) 80:31–43. 10.1007/978-94-017-8881-6_3 24798006

[21] Shokal U Eleftherianos I Evolution and function of thioester-containing proteins and the complement system in the innate immune response. Front Immunol (2017) 8:759 10.3389/fimmu.2017.00759 28706521 PMC5489563

[22] Park SB Aoki T Jung TS. Pathogenesis of and strategies for preventing *Edwardsiella tarda* infection in fish. Vet Res (2012) 43:67. 10.1186/1297-9716-43-67 23035843 PMC3479428

[23] Li MF Sun L Li J. *Edwardsiella tarda* evades serum killing by preventing complement activation via the alternative pathway. Fish Shellfish Immunol (2015) 43:325–39. 10.1016/j.fsi.2014.12.037 25575477

[24] Abreu AG Barbosa AS. How *Escherichia coli* circumvent complement-mediated killing. Front Immunol (2017) 8:452. 10.3389/fimmu.2017.00452 28473832 PMC5397495

[25] Röttgerding F Wagemakers A Koetsveld J Fingerle V Kirschfink M Hovius JW Immune evasion of *Borrelia miyamotoi*: CbiA, a novel outer surface protein exhibiting complement binding and inactivating properties. Sci Rep (2017) 7:303. 10.1038/s41598-017-00412-4 28331202 PMC5428533

[26] Riley SP Patterson JL Nava S Martinez JJ. Pathogenic *Rickettsia* species acquire vitronectin from human serum to promote resistance to complement-mediated killing. Cell Microbiol (2014) 16(6):849–61. 10.1111/cmi.12243 24286496 PMC4028375

[27] Ho DK Jarva H Meri S. Human complement factor H binds to outer membrane protein Rck of *Salmonella*. J Immunol (2010) 185(3):1763–9. 10.4049/jimmunol.1001244 20622116

[28] Wang Z Li MY Peng B Cheng ZX Li H Peng X GC–MS-based metabolome and metabolite regulation in serum-resistant *Streptococcus agalactiae*. J Proteome Res (2016) 15:2246–53. 10.1021/acs.jproteome.6b00215 27251450

[29] Wang Q Yang M Xiao J Wu H Wang X Lv Y Genome sequence of the versatile fish pathogen *Edwardsiella tarda* provides insights into its adaptation to broad host ranges and intracellular niches. PLoS One (2009) 4:e7646. 10.1371/journal.pone.0007646 19865481 PMC2764856

[30] Zeng ZH Du CC Liu SR Li H Peng X Peng B. Glucose enhances tilapia against *Edwardsiella tarda* infection through metabolome reprogramming. Fish Shellfish Immunol (2017) 61:34–43. 10.1016/j.fsi.2016.12.010 27965164

[31] Du CC Yang MJ Li MY Yang J Peng B Li H Metabolic mechanism for L-leucine-induced metabolome to eliminate *Streptococcus iniae*. J Proteome Res (2017) 16(5):1880–9. 10.1021/acs.jproteome.6b00944 28266220

[32] Cheng ZX Ma YM Li H Peng X. N-acetylglucosamine enhances survival ability of tilapias infected by *Streptococcus iniae*. Fish Shellfish Immunol (2014) 40:524–30. 10.1016/j.fsi.2014.08.008 25120218

[33] Yamada T Letunic I Okuda S Kanehisa M Bork P. iPath2.0: interactive pathway explorer. Nucleic Acids Res (2011) 39:W412–5. 10.1093/nar/gkr313 21546551 PMC3125749

[34] Li WX Yao ZJ Sun LN Hu WJ Cao JJ Lin WX Proteomics analysis reveals a potential antibiotic cocktail therapy strategy for *Aeromonas hydrophila* infection in biofilm. J Proteome Res (2016) 15(6):1810–20. 10.1021/acs.jproteome.5b01127 27110028

[35] Chen XH Zhang BW Li H Peng X Myo-inositol improves the host’s ability to eliminate balofloxacin-resistant *Escherichia coli*. Sci Rep (2015) 5:10720 10.1038/srep10720 26030712 PMC5377236

[36] Holers VM. Complement and its receptors: new insights into human disease. Annu Rev Immunol (2014) 32:433–59. 10.1146/annurev-immunol-032713-120154 24499275

[37] Berends ET Kuipers A Ravesloot MM Urbanus RT Rooijakkers SH. Bacteria under stress by complement and coagulation. FEMS Microbiol Rev (2014) 38:1146–71. 10.1111/1574-6976.12080 25065463

[38] Parente R Clark SJ Inforzato A Day AJ. Complement factor H in host defense and immune evasion. Cell Mol Life Sci (2017) 74(9):1605–24. 10.1007/s00018-016-2418-4 27942748 PMC5378756

[39] Hovingh ES van den Broek B Kuipers B Pinelli E Rooijakkers SHM Jongerius I. Acquisition of C1 inhibitor by *Bordetella pertussis* virulence associated gene 8 results in C2 and C4 consumption away from the bacterial surface. PLoS Pathog (2017) 13(7):e1006531. 10.1371/journal.ppat.1006531 28742139 PMC5542704

[40] Sui ZH Li MF Sun L. Tongue sole (*Cynoglossus semilaevis*) CD59: a complement inhibitor that binds bacterial cells and promotes bacterial escape from the killing of fish serum. Fish Shellfish Immunol (2016) 58:442–8. 10.1016/j.fsi.2016.09.051 27688119

[41] Diao J Bouwman C Yan D Kang J Katakam AK Liu P Peptidoglycan association of murein lipoprotein is required for KpsD-dependent group 2 capsular polysaccharide expression and serum resistance in a uropathogenic *Escherichia coli* isolate. MBio (2017) 8(3):e603–17. 10.1128/mBio.00603-17 28536290 PMC5442458

[42] Peng B Su YB Li H Han Y Guo C Tian YM Exogenous alanine or/and glucose plus kanamycin kills antibiotic-resistant bacteria. Cell Metab (2015) 21:249–61. 10.1016/j.cmet.2015.01.008 25651179

[43] Chen XH Liu SR Peng B Li D Cheng ZX Zhu JX Exogenous L-valine promotes phagocytosis to kill multidrug-resistant bacterial pathogens. Front Immunol (2017) 8:207. 10.3389/fimmu.2017.00207 28321214 PMC5337526

[44] Young T Alfaro AC Metabolomic strategies for aquaculture research: a primer. Rev Aqua (2016) 1–31. 10.1111/raq.12146

[45] Young T Kesarcodi-Watson A Alfaro AC Merien F Nguyen TV Mae H Differential expression of novel metabolic and immunological biomarkers in oysters challenged with a virulent strain of OsHV-1. Dev Comp Immunol (2017) 73:229–45. 10.1016/j.dci.2017.03.025 28373065

[46] Dahal N Abdelhamed H Lu J Karsi A Lawrence ML Tricarboxylic acid and one-carbon metabolism pathways are important in *Edwardsiella ictaluri* virulence. PLoS One (2013) 8:e65973 10.1371/journal.pone.0065973 23762452 PMC3676347

[47] Dahal N Abdelhamed H Lu J Karsi A Lawrence ML Effect of multiple mutations in tricarboxylic acid and one-carbon metabolism pathways on *Edwardsiella ictaluri* pathogenesis. Vet Microbiol (2013) 169:107–12. 10.1016/j.vetmic.2013.12.006 24418045

[48] Dahal N Abdelhamed H Karsi A Lawrence ML. Tissue persistence and vaccine efficacy of tricarboxylic acid cycle and one-carbon metabolism mutant strains of *Edwardsiella ictaluri*. Vaccine (2014) 32:3971–6. 10.1016/j.vaccine.2014.05.016 24837777

[49] Fozo EM Scott-Anne K Koo H Quivey RG Jr. Role of unsaturated fatty acid biosynthesis in virulence of *Streptococcus mutans*. Infect Immun (2007) 75:1537–9. 10.1128/IAI.01938-06 17220314 PMC1828593

[50] Schoen C Kischkies L Elias J Ampattu BJ. Metabolism and virulence in *Neisseria meningitidis*. Front Cell Infect Microbiol (2014) 4:114. 10.3389/fcimb.2014.00114 25191646 PMC4138514

[51] McKinney JD Honer zu Bentrup K Munoz-Elias EJ Miczak A Chen B Chan WT Persistence of *Mycobacterium tuberculosis* in macrophages and mice require the glyoxylate shunt enzyme isocitrate lyase. Nature (2000) 406:735–8. 10.1038/35021074 10963599

[52] Munoz-Elias EJ McKinney JD. *Mycobacterium tuberculosis* isocitrate lyases 1 and 2 are jointly required for in vivo growth and virulence. Nat Med (2005) 11:638–44. 10.1038/nm1252 15895072 PMC1464426

[53] Wang H Clouthier SG Galchev V Misek DE Duffner U Min CK Intact-protein-based high-resolution three-dimensional quantitative analysis system for proteome profiling of biological fluids. Mol Cell Proteomics (2005) 4(5):618–25. 10.1074/mcp.M400126-MCP200 15703445

[54] Harden VP Harris JO The isoelectric point of bacterial cells. J Bacteriol (1953) 65(2):198–202. 13034716 10.1128/jb.65.2.198-202.1953 PMC169666

