# Supplementary material for: Edwardsiella tarda Tunes Tricarboxylic Acid Cycle to Evade Complement-Mediated Killing
Source: Front Immunol. 2017 Dec 7;8:1706. doi: 10.3389/fimmu.2017.01706 (PMC5725468; doi:10.3389/fimmu.2017.01706)
Supplement: Supplementary file 1 [file Data_Sheet_1.PDF]

Figure S

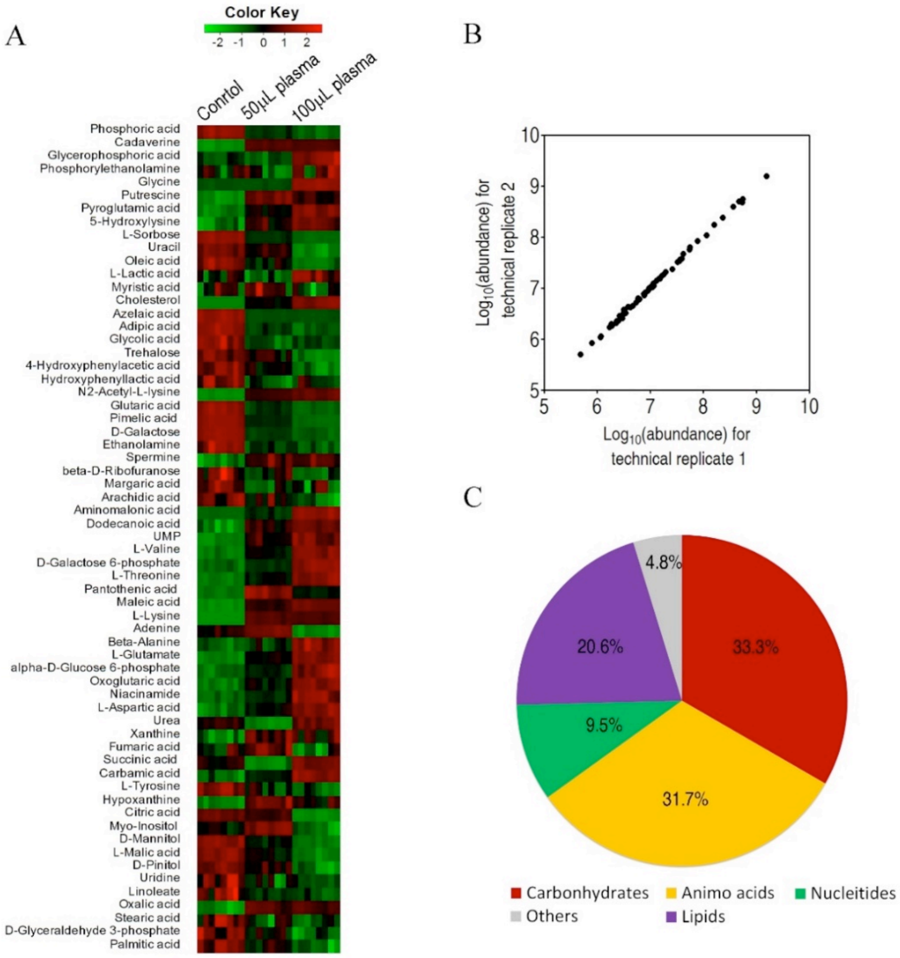

Fig. S1. Metabolic profiles of *E. tarda* in response to serum complement-mediated killing. (A), Heat map of unsupervised hierarchical clustering of total metabolites (row). Red and blue indicate increase and decrease of the metabolites scaled to mean and standard deviation of row metabolite level, respectively (see color scale). **(B)**, Reproducibility of metabolomic profiling platform used in the discovery phase. Abundance of metabolites quantified in samples over two technical replicates is shown. Pearson correlation coefficient between technical replicates varies between 0.994 and 0.999. This plot shows the two replicates with the weakest correlation of 0.994. **(C)**, Classification of metabolites.

Table S

Table S1. QRT-PCR Primers for genes of alanine, aspartate and glutamate metabolism

Gene	F or R	Primers(5'-3')	Primer length (bp)	Products (bp)
16S rRNA	Forward	gtagtccacgctgtaaacga	20	164
	Reverse	gaattaaaccacatgctcca	20	
asnB	Forward	agcgcgactggttcga	16	166
	Reverse	ttggtgatggcggagata	18	
asnA	Forward	aggcgccgatcctgag	16	202
	Reverse	cgcagcgccttcatgt	16	
ansA	Forward	tgccggactttaccatcc	18	255
	Reverse	tggtctgcccgtcagatc	18	
ansB	Forward	ggtgccgcagctgaaaa	17	119
	Reverse	tgcactcggcgttgattt	18	
ETAE_0921	Forward	accgcccacttttggtt	17	242
	Reverse	gccttgagggcgtagatg	18	
ETAE_1150	Forward	gacaagtgcagcggcaac	18	221
	Reverse	gctgataatgcgccgatactc	21	
aspC	Forward	ctccaagaactttggcctgt	20	107
	Reverse	ggatcaccgccttgatctg	19	
ETAE_3480	Forward	tgcgcgaaacctttatcaaa	20	199
	Reverse	gtttcaaagcgcacctgatc	20	
ETAE_3479	Forward	gcttcgccgaggatctg	17	112
	Reverse	gcatccgggttctttttctg	20	
purA	Forward	ttcgccgttaagctgaaag	19	114
	Reverse	ggcaaccgccatcactt	17	
purB1	Forward	atgccggagctgtgtctg	18	112
	Reverse	cgccgagcagatccatatt	19	
purB2	Forward	aaggcggtcgagtacttcct	20	164
	Reverse	cgccagaccggcttaag	17	
gadB2	Forward	tgcgcgaagacattgct	17	12
	Reverse	tatccatcaggcggtgtaca	20	
gabD	Forward	cctggcgcatcaagagg	17	194
	Reverse	cgatgggctgcttgatc	17	
gltB	Forward	aacggcgaaattaacacca	19	120
	Reverse	gccggtttcgttgacaaa	18	
gltD	Forward	tcccgcgtttaaattggaa	19	286
	Reverse	ggagacgtagggggtctgag	20	
ETAE_3353	Forward	agccggagcgagtgatc	17	195
	Reverse	tggtgagcgcatttttaaag	20	
putA	Forward	gaccgcctggagctctct	18	178

	Reverse	tcaccaggcggatcatcag	19	
glnA	Forward	aacccgaccaccaactctt	19	181
	Reverse	cggcgaagcacaggtac	17	
carB	Forward	accgcgaagagtttgaagag	20	115
	Reverse	cgcaccacctccatctcata	20	
carA	Forward	tgaccggctaccaagaaatt	20	123
	Reverse	catgtacggcggaagattct	20	
ybaS	Forward	tcgccctggagtcgat	16	210
	Reverse	ggttgtcggccttaatcag	19	
yneH	Forward	actgcgctttaagaatgaactg	22	252
	Reverse	aaacggcgatgatcatctc	19	
glmS	Forward	cagcgccagggaggtt	16	149
	Reverse	atccctcggccaatcac	17	
aspA	Forward	acgccggaaggctacc	16	227
	Reverse	tccggcaggttgatttc	17	

Table S2. Table S1. QRT-PCR Primers for genes of the TCA cycle

Gene Name	F or R	Primers(5'-3')	Primer length (bp)	Products (bp)
aceE	Forward	cgaagctgatgccgga	16	178
	Reverse	ttggattccggctcatc	17	
aceF	Forward	ctgcgcgaagacgtacag	18	179
	Reverse	ttggcgccggagatct	16	
lpdA	Forward	gcctgaacgtcggctgt	17	133
	Reverse	caggtgcggatcttgtcaa	19	
ETAE_3478	Forward	ctgggcgcagagttttatct	20	266
	Reverse	tcgccggcaataaagatatg	20	
ETAE_1708	Forward	gagcatagcgggcaacat	18	138
	Reverse	gctctgttcggcgatctg	18	
gltA	Forward	accatgactcgctggaca	18	214
	Reverse	tccagcaccgggttgac	17	
acnB	Forward	ctgattgaggcgctgga	17	154
	Reverse	catcggcccaggactg	16	
ETAE_2503	Forward	agcccgaacctgacgatc	18	148
	Reverse	gacttgcgcgagtcatagac	20	
ETAE_2050	Forward	cgtgaaaacgccgaggat	18	152
	Reverse	tcggagcacggcttcac	17	
sucA	Forward	tctcgacccggcgttt	16	253
	Reverse	cagaaagcggcgcttctc	18	
sucB	Forward	gcccttgacggcgaag	16	253
	Reverse	tgataatcggcgtggacatc	20	
sucC	Forward	aggcccagcagtttggtc	18	248
	Reverse	cagcgcgacgtagttcag	18	
sucD	Forward	ggcgatagacggcggta	17	137
	Reverse	tgatcacccccggacag	17	
sdhA	Forward	ccaccaacgcccacatc	17	267
	Reverse	ccttcgcggatctctatcat	20	
sdhB	Forward	cggaagaggggcgtgac	17	133
	Reverse	ttttggccgttcatgttga	19	
sdhC	Forward	ccaacaatccgctttcctg	19	217
	Reverse	ccgccgcagatgtgatag	18	
sdhD	Forward	cgcatccgccatcatc	16	112
	Reverse	ggtgatccgcgaagagaa	18	
frdA	Forward	agcgtctgccgttcatct	18	150
	Reverse	cgaacaggcccttgatgc	18	
frdB	Forward	tggccaacttcccggtt	17	174
	Reverse	agttgatgcagccggagaac	20	
frdC	Forward	ttcctgcaaaacccgct	17	157

	Reverse	cccacagcgccttgataatc	20	
frdD	Forward	gcggtatgtggggagct	17	166
	Reverse	cacagcggcagaatgatc	18	
fumC	Forward	gcgcagatcgccgaac	16	262
	Reverse	tggtcagcgcctcacact	18	
ETAE-2114	Forward	ctgctcggcggatcgta	17	243
	Reverse	gcgtgggcgatatcacg	17	
mdh	Forward	ccggtgattggcggtc	16	253
	Reverse	tctcgacgtagccgcactc	19	
